# Effects of Duloxetine Treatment on Cognitive Flexibility and BDNF Expression in the mPFC of Adult Male Mice Exposed to Social Stress during Adolescence

**DOI:** 10.3389/fnmol.2016.00095

**Published:** 2016-10-04

**Authors:** Hang Xu, Yu Zhang, Fan Zhang, San-na Yuan, Feng Shao, Weiwen Wang

**Affiliations:** ^1^CAS Key Laboratory of Mental Health, Institute of PsychologyBeijing, China; ^2^School of Humanities, The University of Chinese Academy of SciencesBeijing, China; ^3^School of Nursing, Binzhou Medical UniversityYantai, China; ^4^Beijing Key Laboratory of Behavior and Mental Health, School of Psychological and Cognitive Sciences, Peking UniversityBeijing, China

**Keywords:** social stress, adolescent, cognitive flexibility, medial prefrontal cortex (mPFC), brain-derived neurotrophic factor (BDNF)

## Abstract

Early stress is a significant risk factor for the onset of mood disorders such as depression during adulthood. Impairments in cognitive flexibility mediated by prefrontal cortex (PFC) dysfunction are increasingly recognized as important etiological and pathological factors in the development of depression. Our previous study demonstrated that social defeat stress during early adolescence produced delayed deficits in cognitive flexibility in adult mice. The potential molecular mechanisms underlying these long-term consequences remain unclear. One candidate molecule is brain-derived neurotrophic factor (BDNF), which plays a vital role in neural development and synaptic plasticity. In this study, we initially examined the effects of adolescent social stress on cognitive flexibility and PFC BDNF expression within a week after the last stress exposure and 6 weeks later during adulthood. Adolescent (PND 28) male mice were subjected to stress or control manipulation for 10 days. The attentional set-shifting task (AST) was used to assess cognitive flexibility. Levels of BDNF mRNA and protein in the PFC were examined after behavioral testing. The results demonstrated that previously stressed mice exhibited delayed extra-dimensional set-shifting deficits in AST when tested as adults but not when tested as adolescents. Consistent with the cognitive alterations, adolescent stress induced dynamic alterations in BDNF expression in the medial PFC (mPFC), with a transient increase observed shortly after the stress, followed by a decrease 6 weeks later during adulthood. Next, we further determined the effects of chronic treatment with the antidepressant duloxetine during early adulthood on cognitive and molecular alterations induced by adolescent stress. Compared with the controls, duloxetine treatment reversed the cognitive deficits and increased the BDNF protein expression in the mPFC during adulthood in previously stressed mice. These findings demonstrated that BDNF expression in the mPFC was sensitive to adolescent social stress, which may contribute to the disturbance of the development and later functioning of this brain region.

## Introduction

Cognitive flexibility, in which the prefrontal cortex (PFC) plays an integrative role, is a basic ability to consistently adapt to a dynamic environment using appropriate behavioral strategies (Robbins, 2007). Impairments in cognitive flexibility are increasingly recognized as a major component of depression, as described in etiological, pathophysiological and pharmaceutical studies (Lapiz-Bluhm et al., 2008). For example, patients with depression frequently exhibit deficits in cognitive flexibility, and these deficits are related to the duration and severity of the depression symptoms (Millan et al., 2012; Peters et al., 2015). The impaired ability to shift attentional set from negative affective dimension in response to changing environmental conditions is an important predisposing factor in the onset of depression (Marazziti et al., 2010; Goeldner et al., 2013). The effects of antidepressant treatment on cognitive dysfunction can predict the recurrence of depression (Harmer et al., 2009; Chang et al., 2012).

Adversely stressful experiences during early life have been identified as major risk factors for the development of depression in later life (Lupien et al., 2009). Bullying and subordination are ethologically relevant social stressors that are prevalent in adolescents. Bullying during adolescence can cause long-lasting behavioral and neural consequences that closely connect with depression in adulthood (Newman et al., 2005). The social defeat stress model in rodents has been utilized to reflect such stressful experiences in humans (Miczek, 1979; Golden et al., 2011). Consistently, rodent studies have also shown that adolescent social stress can induce a set of depressive behaviors during adulthood, including anhedonia, locomotor activity in the open field test, anxiety in the elevated plus maze and the object-burying test and social avoidance of the defeat context (Buwalda et al., 2011; McCormick and Green, 2013). Recent studies from our laboratory and by Snyder et al. (2015) reported that adolescence was a sensitive time window in which social defeat could induce deficits in set shifting in adult animals under the experimental conditions used (Zhang et al., 2016).

Successful performance of cognitive flexibility depends on the normal structure and function of the PFC (Dalley et al., 2004). Lesion and chronic stress studies have shown that reversal learning (RL) and extra-dimensional set shifting (EDS) are specifically mediated by the orbitofrontal cortex (OFC) and the medial PFC (mPFC), respectively (Birrell and Brown, 2000; McAlonan and Brown, 2003; Floresco et al., 2008). There is a significant decrease in neuroplasticity of the mPFC in depression, and chronic antidepressant treatment can exert therapeutic effects via the ability to enhance neuroplasticity (Duman and Monteggia, 2006; Pompili et al., 2013). These studies suggest that the set-shifting impairments in adulthood seen in our previous study may be attributed to the damage of the mPFC resulting from the exposure to adolescent social stress. As a brain area that undergoes late maturation, the PFC and relevant pathways exhibit significant reorganization during adolescence, characterized by both progressive and regressive changes including alterations in gray matter thickness and synapse and receptor density (Spear, 2000; Andersen and Teicher, 2008). In addition, the main stress system, the hypothalamic-pituitary-adrenal axis, is still immature in adolescence and responses to stressors are enhanced or more persistent during adolescence than in adulthood (McCormick and Mathews, 2010). Exposure to stress during this sensitive period may alter the developmental pattern of the PFC and lead to the cognitive consequences mentioned above. Previous studies support the idea that one long-term outcome of adolescent adversity is the reduction of structural and functional plasticity in the PFC, which is consistent with an increased likelihood of developing depression in adulthood (Eiland et al., 2012).

Neurotrophic factors, particularly the brain-derived neurotrophic factor (BDNF), have important roles in the pathophysiology of depression and in the therapeutic effects of antidepressant treatment (Duman and Monteggia, 2006; Castrén and Rantamäki, 2010; Hashimoto, 2015). Additionally, BDNF is intimately involved in neural development, synaptic plasticity and neurogenesis in brain areas that mediate learning and memory, especially the PFC and hippocampus (Yamada and Nabeshima, 2003; Ninan, 2014). Early stress has been reported to induce lasting alterations in BDNF expression in the PFC and hippocampus in adult animals, depending on the experimental conditions, including age at exposure to stressors, form and duration of the stressor and gender (Cirulli et al., 2009; Bath et al., 2013). In addition, postnatal BDNF levels vary significantly during development in a regionally specific manner, suggesting that BDNF differentially affects the maturation of specific brain structures (MacQueen et al., 2003; Yeh et al., 2012). For example, in rodents, PFC BDNF rapidly increases and peaks in early adolescence (postnatal day (PND) 28) and then gradually decreases to adult levels (Kolbeck et al., 1999; Bath et al., 2013). The developmental pattern of BDNF expression during adolescence is similar to that of the PFC structure and function mentioned above. Thus, exposure to stress during this stage may disturb the developmental profile of BDNF, inducing persistent and deleterious effects on PFC development and function later in life.

These findings and our previous study demonstrate that early adolescence (PND 28–37) is a critical period in which social defeat stress can induce a delayed deficit in cognitive flexibility in adulthood (Zhang et al., 2016). Therefore, the first aim of this study was to determine whether BDNF expression in the PFC was altered along with the cognitive changes following adolescent social defeat. Parallel to the time course of cognitive deficit development, PFC BDNF expression was quantified 1 week after the stress and 6 weeks later during adulthood. Furthermore, we examined whether adult alterations in cognition and BDNF expression that were induced by adolescent stress could be normalized by pharmacological intervention during an early stage before behavioral testing. Noradrenergic and serotonergic systems in the PFC have been implicated in the modulation of cognitive flexibility, which can be affected by social stress and antidepressants with actions targeted at these systems (Robbins and Arnsten, 2009; Buwalda et al., 2011). Moreover, normalized or ameliorated BDNF inhibition in the hippocampus and PFC is required for the behavioral effects of antidepressants in rodents (Duman and Monteggia, 2006; Ball et al., 2013; Engel et al., 2013; Kozisek et al., 2008). Thus, the ability of duloxetine (a dual noradrenaline/serotonin reuptake inhibitor) to relieve changes in cognition and BDNF expression in previously stressed mice during adulthood was examined.

## Materials and Methods

### Animals

Male offspring of C57BL/6J mice (the Academy of Chinese Military Medical Science) were used as the intruder subjects and obtained at weaning (PND 21) from our in-house breeding program (Center of Experimental Animal, Institute of Psychology, Chinese Academy of Sciences). At PND 21, male siblings were housed in groups of 2–4 mice/cage until the start of the experiment. Adult (2–2.5 months old) CD1 mice (Vital River Laboratories) were used as the resident subjects and housed individually. All animals were kept under controlled environmental conditions (ambient temperature 20 ± 2°C, 12 h light–dark cycle with lights on at 07:00 a.m.) with free access to water and food except during the attentional set-shifting task (AST). All experimental procedures were approved by the Institutional Review Board of the Institute of Psychology, Chinese Academy of Sciences and were in compliance with the National Institutes of Health Guide for the Care and Use of Laboratory Animals.

### Experimental Procedures

The Experiment was Conducted in Two Parts:

Experiment 1: at PND 28, male siblings were separately assigned to two groups: stress (STR) or control (CON). Mice in the stress and CON groups were exposed to social defeat stress or manipulated for 10 days (PND 28–37). After that, all mice were individually housed. Some mice in each group underwent behavioral testing 1 week after the last stress session. Twenty-four hours after the behavioral assessment, the mice were sacrificed and brain samples were collected. Other mice were individually housed until 6 weeks later during adulthood (PND 80), for the second test; the experimental procedure was replicated. Single housing after the stress is necessary for the adult deficit of cognitive function induced by social defeat during early adolescence, and this effect can be ameliorated by social housing (with siblings) after the stress (for details, see Zhang et al., 2016).

Experiment 2: the stressed and CON groups were similar to those described for experiment 1. Four weeks after the last stress, mice in the STR and CON groups were further divided into two subgroups: saline (SAL) or duloxetine (DUL). They were intraperitoneally injected with saline or duloxetine at a volume of 0.1 ml/25 g body weight daily over a period of 14 days (PND 65–79). At PND 80, the same behavioral tests and brain sampling procedures outlined for experiment 1 were performed.

### Drug Administration

Duloxetine [(S)-Duloxetine Hydrochloride] (Sigma-Aldrich, St. Louis, MO, USA) used in experiment 2 was dissolved in saline solution (0.9%) at a concentration of 2.5 mg/ml. Beginning at PND 65, stress and CON mice were treated with duloxetine or saline at a dose of 10 mg/kg for 14 days. This dose was chosen based on the stable antidepressant action of duloxetine reported in previous studies (Calabrese et al., 2007; Engel et al., 2013).

### Social Stress

The “resident-intruder” paradigm similar to that in previous studies by us and others was performed to mimic social defeat (Golden et al., 2011; Zhang et al., 2016). In brief, a daily stress protocol consisted of two steps: first, the intruder C57BL/6J mouse in the defeat group was placed in the cage of the resident CD-1 mouse and was attacked for 5 min or for 3 min if the attack by the CD-1 mouse was intense (attack latency shorter than 60 s, multiple or continuous attack). Thereafter, the intruder was separated from the resident by a clear perforated Plexiglas divider; sensory contact with the resident was maintained for the remainder of the 24 h period. The C57BL/6J intruders were rotated every day across nine defeat days to avoid habituation to a single aggressor. CON mice were pair housed with other C57BL/6J mice but kept on the opposite side of the cage by the divider. All CON mice were rotated on a daily basis in a manner similar to that of mice undergoing defeat, but they were never allowed physical contact with their cage mate.

### Behavioral Tests

On the last day of the social defeat or control manipulation, the mice were placed on food restriction for 7 days to maintain 80–85% original body weight with free access to water. AST testing was similar to the method described for a previous study (Bissonette and Powell, 2012; Yuan et al., 2014). In brief, a 4-day training and testing protocol was initiated on the fourth day of food restriction. The mice were initially trained to locate a buried reward by digging into two sawdust-filled pots placed in the home cage. On the fifth day, mice were transferred to the testing arena and trained to retrieve the reward from both sawdust-filled pots. On the sixth day of food restriction, mice were required to perform two separate simple discrimination (SDs) using two sets of exemplar pots scented with different odors (lemon vs. rosewood, both pots filled with sawdust) or filled with different digging media (foam sheets vs. shredded paper, no odor). On the seventh day of testing, a five-stage discrimination task, including SD, compound discrimination (CD), intra-dimensional shifting (IDS), RL and extra-dimensional set shifting EDS were performed. Animals proceeded from one stage to the next after the criterion of six consecutive correct trials was achieved. A typical task sequence and a description of the test stimuli are included in Supplementary Table S1. The number of trials to achieve the criterion and the number of errors to achieve the criterion were recorded at each stage for each mouse.

### RNA Extraction for qPCR and Analysis of BDNF mRNA

For qPCR, the mice were decapitated 24 h after the end of behavioral testing, and the brain was quickly removed. The mPFC was rapidly dissected on an ice plate pretreated with RNA stabilization solution. The tissue was immediately frozen in liquid nitrogen and then stored at –80°C for subsequent analysis. In particular, according to the atlas of Paxinos and Franklin (2003), the mPFC was dissected from a 1-mm-thick coronal section using a mouse brain mold (approximately 2.34–1.34 mm from the bregma, containing the cingulate, prelimbic and infralimbic (IL) subregions) under microscopic observation (Leica, Germany).

Total RNA was isolated by homogenization in TRIzol Reagent (Invitrogen, Carlsbad, CA, USA), according to the manufacturer’s instructions. The concentration and purity of the isolated RNA were determined by measuring the absorbance (NanoDrop 2000, Thermo, Wilmington, DE, USA). Next, cDNA was prepared from RNA with the SuperScript^TM^ III reverse transcription kit (Invitrogen, USA) according to the manual. The following forward (F) and reverse (R) primers (Invitrogen) were used in this study. For GAPDH, F: TGCACCACCAACTGCTTA; R: AAGTGTACAAGTCCGCGTCC; for BDNF, F: TGGCTGACACTTTTGAGCAC; R: AAGTGTACAAGTCCGCGTCC. The housekeeping gene GAPDH was used as an internal CON and was amplified from each sample. Real-time quantitative PCR using cDNA from individual animals was performed. The amplification protocol was as follows: 95°C for 10 min, 95°C for 30 s and 60°C for 1 min (40 cycles), followed by signal detection for 10 s. Each sample was assessed in duplicate, and the average value was calculated for each sample. Relative BDNF mRNA expression levels were calculated according to the 2(−ΔΔC (T)) method.

### Protein Extraction and Western Blot Analysis of BDNF Protein Levels

The target tissue, mPFC, was collected as described for qPCR analysis and homogenized in lysis buffer supplemented with protease inhibitor cocktail (Roche Diagnostics, Indianapolis, IN, USA). The protein concentration was determined using a BCA Protein Assay Kit (Cwbiotech, Beijing, China). Equal amounts of protein were added with loading buffer and denatured at 95°C for 8 min before separation by SDS-PAGE (12% polyacrylamide gel). After transfer to a PVDF membrane (Millipore, Bedford, MA, USA), nonspecific binding sites were blocked with 5% fat-free milk for 1 h at RT. The membranes were subsequently incubated overnight at 4°C with a primary rabbit polyclonal anti-BDNF antibody (1:1000, Abcam, Cambridge, UK) and a mouse β-actin antibody (1:1000, TA-09, Zhongshan Golden Bridge, Beijing, China). After being washed in TBST (3 × 10 min), the membranes were incubated with an HRP-conjugated goat anti-rabbit IgG secondary antibody (1:10,000, Jackson ImmunoResearch, West Grove, PA, USA) for 40 min at RT and then washed again. The images were visualized with enhanced chemiluminescence (ECL, Millipore, Bedford, MA, USA) using a FluorChem E System (Protein Simple, Santa Clara, CA, USA). Quantity One software (Bio-Rad, Hercules, CA, USA) was used to quantify the data to determine protein levels, which were normalized to the levels of β-actin.

### Immunohistochemical Analysis of BDNF Protein Levels

**Figure 1:**
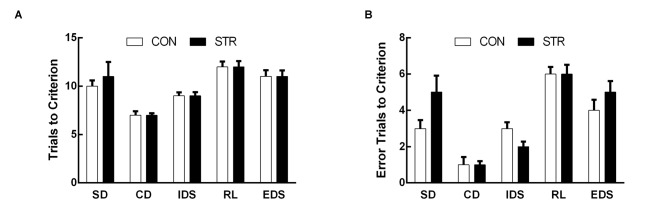
**Short-term effects of social stress (STR) during early adolescence on the performance on the attentional set-shifting task (AST).** Mice were tested 1 week after the last stress. Bars indicate the mean number of trials to criterion **(A)** and errors to criterion **(B)** for the simple discrimination (SD), compound discrimination (CD), intra-dimensional shifting (IDS), reversal learning (RL) and extra-dimensional shifting (EDS) stages of the AST. *n* = 10 mice/group.

The protocols were adapted from our previous studies (Wang et al., 2015). Twenty-four hours after the final behavioral test, the mice were deeply anesthetized with 10% chloral hydrate (0.3 ml, intra-peritoneal injection) and transcardially perfused with pre-cooled (4°C) 0.1 M PBS followed by 4% paraformaldehyde in 0.1 M PBS (pH 7.4). Brains were removed and post-fixed in 0.01 M PBS containing 4% PFA for 24 h. A 4-mm segment of interest containing mPFC (3.56–0.72 mm from the bregma) was dissected using a mouse brain mold. The samples were dehydrated, cleared and embedded in paraffin at 60°C for later analysis. Prior to analysis, the samples were sectioned on a microtome (Leica Microsystems, Wetzlar, Germany) at RT, and coronal paraffin sections (5 μm thick) containing the target area of mPFC (1.98–1.34 mm from the bregma, Figure 1) were prepared (Paxinos and Franklin, 2003). The sections were transferred onto coated slides (2 sections/slide) and dried on a heating plate at 60°C for 30 min.

For immunohistochemical analysis, the sections were dewaxed 3 × 10 min in xylene and then dehydrated by serial ethanol rinsing (2 × 2 min in 100% ethanol, 2 × 2 min in 95% ethanol and 1 × 2 min in 85% ethanol). After washing in 0.01 M PBS (3 × 2 min), the sections were heated in a 0.1 M citrate buffer solution in a microwave oven. The sections were then washed again in 0.01 M PBS (3 × 2 min), incubated in 3% hydrogen peroxide (15 min at RT) to eliminate endogenous peroxidase and then washed again and incubated in a blocking solution (0.01 M PBS with 5% goat serum) for 25 min at RT. The sections were then incubated with primary rabbit anti-BDNF polyclonal antibody (1:200, Santa Cruz Biotechnology, CA, USA) overnight at 4°C, washed in 0.05 M PBS (3 × 5 min), incubated in secondary goat anti-rabbit antibody (1:1000, Santa Cruz Biotechnology, CA, USA) for 1.5 h at RT, and then washed again. Finally, the sections were stained with 0.05% 3,3′-diaminobenzidine (DAB; ZLI-9018, Zhongshan Golden Bridge, Beijing, China), dehydrated and transparentized by gradient ethanol (1 × 2 min 85%, 2 × 2 min 95%, 2 × 2 min 100%) and xylene treatment (3 × 10 min), and then cover-slipped with neutral balsam.

Quantification of the staining density (optical density (OD)) was performed in a blinded manner using a light microscope (Olympus BX-51 with a Camedia Master C-3040 digital camera) and ImageJ software (version 1.37 V, NIH, Bethesda, MD, USA). For each subject, the staining intensity in the mPFC was analyzed. Each region of interest (ROI) within the mPFC was analyzed in three consecutive sections. For each section, the staining density of nonspecific background labeling was measured in a cell-free area and digitally subtracted so that any background staining was eliminated. The ROIs for mPFC remained uniform for each section. For analysis, OD values of the three bilateral sections were averaged into a single score for each animal.

### Statistics

The commercially available program SPSS 16.0 was used for the statistical analysis. All data are presented as the mean ± standard error of the mean (M ± SEM). The AST data analysis was performed using two-way ANOVA (stress × stage) with repeated measures in experiment 1 and three-way ANOVA (stress × drug × stage) with repeated measures in experiment 2. The BDNF mRNA and protein data from experiment 1 were analyzed using the *t*-test, and the BDNF immunochemistry data in experiment 2 were analyzed using two-way ANOVA (stress × drug). When significant main effects or interactions were detected, *t*-test or LSD *post hoc* comparisons were conducted. Significance for all analyses was set at *p* < 0.05.

## Results

To assess the validity of the AST task, data for each AST test in the CON mice in two experiments were used to analyze the effect of the stage. The response pattern across stages, that is, the relatively increased difficulty in the RL and EDS stages reflects the validity of the set-shifting manipulation (Birrell and Brown, 2000; Lapiz-Bluhm et al., 2008). Consistently, this study demonstrated that a significant main effect of stage existed for each AST performance in the CON mice, which was indicated by the increased numbers of trials and/or errors to the criterion in the RL or EDS stages compared with the other stages. These findings validate the AST used in this study. To avoid repetition, the validity analysis was not discussed in the subsequent section.

### Experiment 1: Short-Term Effects of Adolescent Social Stress on AST Performance and BDNF Expression

#### AST

When AST was tested during the week following stress exposure, the trials to criterion data demonstrated significant a main effect of stage (*F*_(4,72)_ = 18.683, *p* < 0.0001) but not of stress (*F*_(1,18)_ = 0.141, *p* > 0. 05), or of the stage × stress interaction (*F*_(4,72)_ = 0.723, *p* > 0.05; Figure 1A).

For errors to criterion, there was a significant main effect of stage (*F*_(4,72)_ = 26.680, *p* < 0.0001) but not of stress (*F*_(1,18)_ = 0.029, *p* > 0.05), or of the stage × stress interaction (*F*_(4,72)_ = 1.313, *p* > 0.05; Figure 1B).

#### Levels of BDNF mRNA in the mPFC

**Figure 2:**
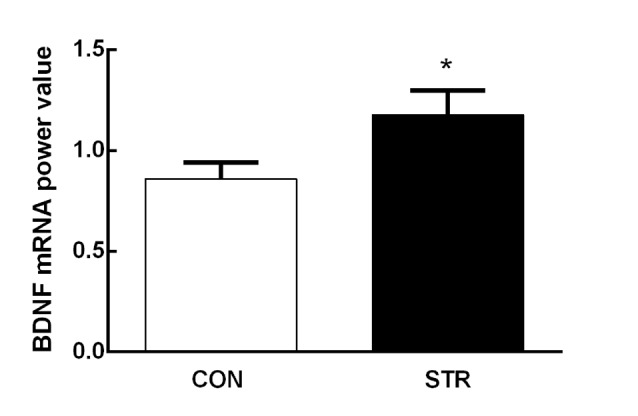
**Short-term effects of social stress during early adolescence on the expression of brain-derived neurotrophic factor (BDNF) mRNA in the medial prefrontal cortex (mPFC).** The BDNF mRNA expression was assessed 24 h after the end of the AST test, which was performed 1 week after the last stress. *n* = 10 mice/group. *p < 0.05 compared with the controls controls (CON).

Adolescent social stress induced an increase in mPFC BDNF mRNA expression compared with the controls 1 week after the last stress (*t*_(1,18)_ = −2.128, *p* < 0.05; Figure 2).

### Experiment 1: Long-Term Effects of Adolescent Social Stress on AST Performance and BDNF Expression

#### AST

**Figure 3:**
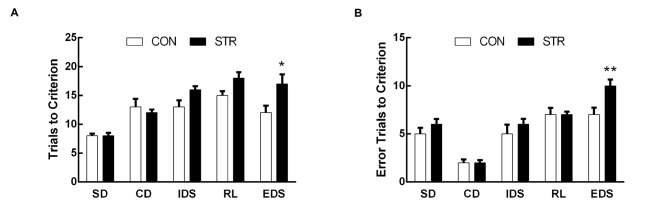
**Long-term effects of social stress during early adolescence on the performance of AST.** Mice were tested 6 weeks after the last stress. Bars indicate the mean number of trials to criterion **(A)** and errors to criterion **(B)** for the SD, CD, IDS, RL and EDS stages of the AST. *n* = 13 in the CON group and *n* = 16 in the stress group. *p < 0.05, **p < 0.01 compared with the controls.

For the trials to criterion tested during adulthood, there were significant main effects of stage (*F*_(4,108)_ = 19.411, *p* < 0.0001) and stress (*F*_(1,278)_ = 10.509, *p* < 0.01) but not of the stage × stress interaction (*F*_(4,108)_ = 1.251, *p* > 0.05; Figure 3A). Further, *t*-tests for each stage showed that socially stressed mice required significantly more trials to criterion in the EDS stage than did the corresponding CON mice (*p* < 0.05). There was no significant difference between the control and stressed groups in the performance of other stages of the AST.

For errors to criterion that were conducted 6 weeks later during adulthood, there were significant main effects of stage (*F*_(4,108)_ = 91.762, *p* < 0.0001), stress (*F*_(1,27)_ = 8.550, *p* < 0.01) and the stage × stress interaction (*F*_(4,108)_ = 3.899, *p* < 0.01; Figure 3B). Then, *t*-tests for each stage showed that socially stressed mice exhibited significantly more error trials to criterion in the EDS stage than did the corresponding CON mice (*p* < 0.01).

### Levels of BDNF mRNA and Protein in the mPFC

**Figure 4:**
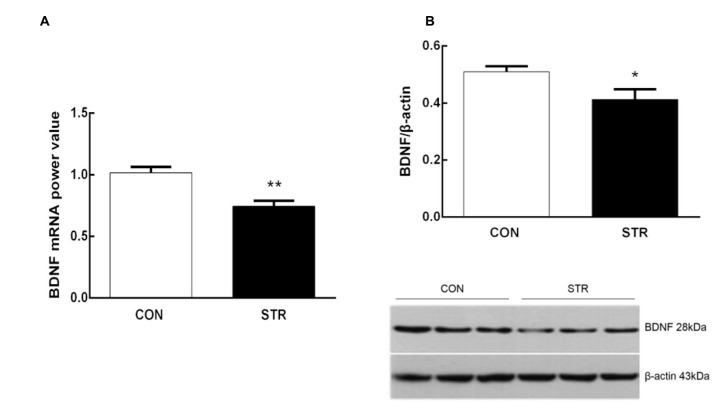
**Long-term effects of social stress during early adolescence on BDNF mRNA levels and protein expression in the mPFC.** The BDNF mRNA **(A)** and protein **(B)** levels in the mPFC were determined 24 h after the AST test, which was conducted 6 weeks after the last stress. *n* = 7–9 mice/group for PCR analysis, *n* = 6–7 mice/group for western blot analysis. *p < 0.05, **p < 0.01 compared with the controls.

As shown in Figure 4A, when tested 6 weeks after the last stress, adult mice that experienced social stress during adolescence exhibited lower levels of BDNF mRNA in the mPFC than the adult controls (*t*_(1,14)_ = 4.103, *p* < 0.01). In addition, western blot analysis showed that previously stressed mice had less BDNF protein in the mPFC than the corresponding controls during adulthood (*t*_(1,11)_ = 2.433, *p* < 0.05; Figure 4B).

### Experiment 2: Effects of Duloxetine Treatment During Early Adulthood on Cognitive Alteration and on BDNF Expression in Adult Mice Subjected to Adolescent Social Stress

#### AST

**Figure 5:**
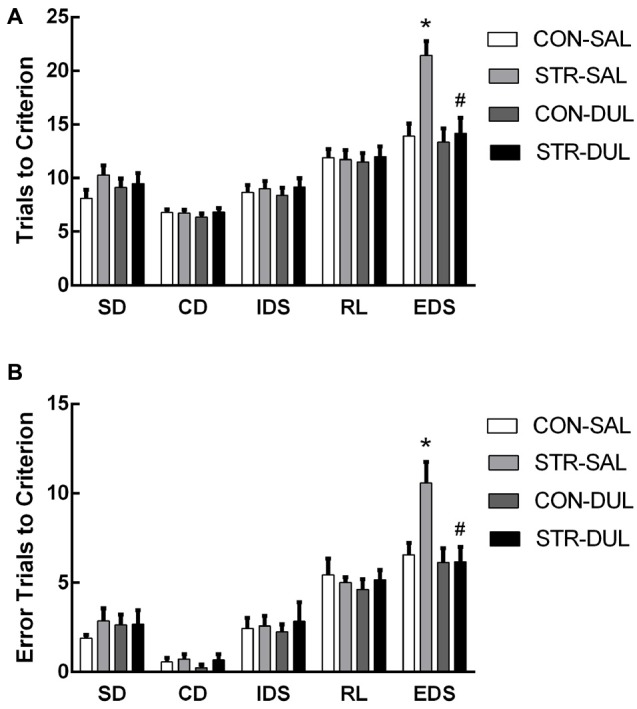
**Effects of duloxetine treatment on cognitive deficits in adult mice submitted to adolescent social stress.** Mice were treated with duloxetine for 2 weeks during early adolescence (10 mg/kg, daily i.p. injection during PND 66–79). Bars indicate the mean number of trials to criterion **(A)** and errors to criterion **(B)** for the SD, CD, IDS, RL and EDS stages of the AST. *n* = 6–9 mice/group. *p < 0.05 compared with the CON-Saline (SAL) group, ^#^*p* < 0.05 compared with the stress-SAL group.

For trials to criterion, there were significant main effects of stage (*F*_(4,104)_ = 53.220, *p* < 0.0001) and stress (*F*_(1,26)_ = 10.459, *p* < 0.01) but not drug (*F*_(1,26)_ = 2.833, *p* > 0.05). More importantly, there were significant interactions of stage × stress (*F*_(4,104)_ = 4.075, *p* < 0.05) and stage × stress × drug (*F*_(4,104)_= 3.383, *p* < 0.05; Figure 5A). Subsequent *post hoc* ANOVA for all task stages revealed significant stress effects for EDS (*F*_(3,26)_ = 7.413, *p* < 0.01). *Post hoc* comparisons demonstrated that adults that were stressed during early adolescence exhibited higher numbers of trials to criterion for EDS than CON mice and duloxetine treatment reversed this change (*p* < 0.05).

For errors to criterion, there were significant main effects of stage (*F*_(4,104)_ = 70.832, *p* < 0.001) but not of stress (*F*_(1,26)_ = 0.560, *p* > 0.05) or drug (*F*_(1,26)_ = 0.787, *p* > 0.05). In addition, there were significant interactions of stage × drug (*F*_(4,104)_ = 2.559, *p* < 0.05) and stage × stress × drug (*F*_(4,104)_ = 2.847, *p* < 0.05; Figure 5B). Subsequent *post hoc* ANOVA for all task stages revealed significant stress effects for EDS (*F*_(3,26)_ = 4.724, *p* < 0.01). *Post hoc* comparisons demonstrated that adults that were stressed during early adolescence exhibited higher numbers of errors for EDS than CON mice and duloxetine treatment reversed this change (*p* < 0.05).

#### Levels of BDNF Protein Levels in the mPFC

**Figure 6:**
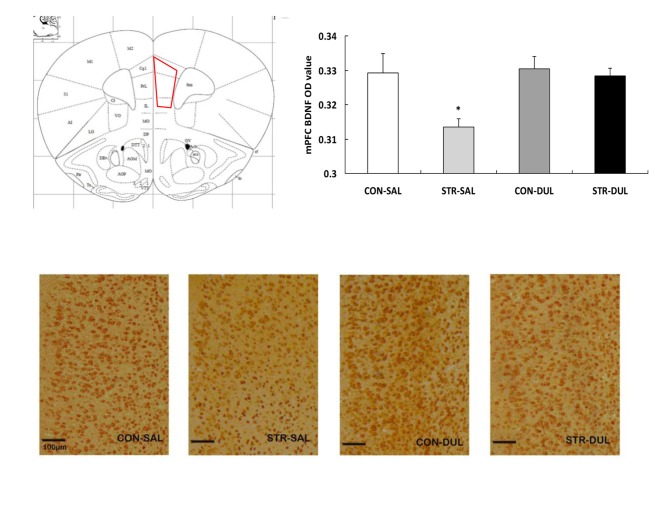
**Effects of duloxetine treatment on regional expression of BDNF protein in adult mice submitted to adolescent social stress.** Representative coronal section of the mPFC adapted from Paxinos and Franklin (2003) and a region of interest (ROI) in which the staining density was quantified. ROI of the mPFC encompasses the prelimbic (PrL), infralimbic (IL) and cingulate (Cg) subareas. Bars represent the mean optical density (OD) value of BDNF immunoreactive profiles in the mPFC (*n* = 6–9 mice/group). *p < 0.05 compared with the CON-SAL and stress-DUL groups.

Significance differences in BDNF expression in the mPFC were observed between groups (*F*_(3,36)_ = 3.166, *p* < 0.05; Figure 6). *Post hoc* analysis revealed that mPFC BDNF protein expression in the stress-SAL group was significantly lower than that in the CON-SAL group (*p* < 0.05). Chronic duloxetine administration ameliorated this decrease, as demonstrated by the increase in BDNF expression in the stress-DUL group compared with the stress-SAL group (*p* < 0.05).

To further assess regional effects of adolescent social stress, BDNF expression in the OFC (including the lateral, ventral and medial sub-regions of the OFC) was also analyzed. There were no main effects of stress (*F*_(1,26)_ = 1.147, *p* > 0.05), drug (*F*_(1,26)_ = 0.107, *p* > 0.05), or interaction of stress × drug (*F*_(1,26)_ = 1.128, *p* > 0.05) on BDNF expression in the OFC (data not shown).

## Discussion

Adversity during childhood and adolescence, such as bullying and abuse, can result in greater susceptibility to depression during adulthood (Gladstone et al., 2006; McCormick and Green, 2013). Using an etiological social defeat stress model, we evaluated the time course of alterations in cognitive flexibility and BDNF expression in the PFC induced by stress during adolescence. Consistent with our previous study, this study confirmed that adolescent social stress induced a delayed expression of set-shifting impairment in mice as adults but not as adolescents (Zhang et al., 2016). Furthermore, such developmental changes in cognitive dysfunction were accompanied by alterations in BDNF expression in the mPFC, but not in the OFC. These behavioral and molecular alterations in previously stressed mice were reversed by chronic treatment with the antidepressant duloxetine during early adulthood. Considering the critical role of BDNF in the neural development and synaptic plasticity underlying cognitive function, the current findings suggest that the mPFC is a sensitive area that mediates the cognitive and molecular consequences of adolescent social stress.

### Effects of Adolescent Social Stress on AST Performance in Mice

In adult animal, lesions, pharmaceutical and chronic stress studies have demonstrated that the mPFC plays a critical role in set-shifting performance (Birrell and Brown, 2000; McAlonan and Brown, 2003; Liston et al., 2006). This suggested that impaired EDS performance in the AST, as seen in this and the previous study after adolescent social stress, might be attributable to abnormal mPFC functioning. In rodents, significant structural and functional remodeling of the mPFC occurs throughout the adolescence, with progressive and regressive changes in synapses and receptor (Giedd, 2008). It is thought that at this stage, neural structures undergoing rapid development are more susceptible to stress, which may alter the trajectories of critical developmental events, resulting in the expression of behavioral symptoms after their maturation in adulthood (Andersen and Teicher, 2008). Consistent with this, several studies have indicated that adolescent stress, particularly early adolescent stress, can cause long-term changes in synaptic density and morphology of the mPFC in adult animals (Leussis and Andersen, 2008; Eiland et al., 2012). This study provided additional evidence that alterations in mPFC function mediated the impaired cognitive performance induced by adolescent social defeat stress. Moreover, the data suggest that the mPFC alterations develop over time as they were not present 1 week after the social stress.

### Effects of Adolescent Social Stress on BDNF Expression in Mice

In parallel with the development of cognitive dysfunction after adolescent social defeat exposure, there had corresponding changes in mPFC BDNF expression in this study. Brain BDNF expression can be affected by multiple factors, including age and stress (Fanous et al., 2010; Taylor et al., 2011; Bath et al., 2013). In the rodent, PFC BDNF expression initially changes in a regionally specific manner across postnatal developmental stages, characterized by very low levels during prenatal and neonatal periods, then a rapid increase to peak levels during pre- and early adolescence, followed by a gradual decline over the lifespan (Kolbeck et al., 1999; Bath et al., 2013; Luoni et al., 2014). This study showed there was no difference in the mPFC BDNF mRNA levels between CON mice at PND 45 and at PND 87 (*t*_(1,16)_ = 0.802, *p* = 0.434, data not shown). This result is consistent with findings by Guo et al. (2010), who reported that the protein expression of BDNF in the rat PFC was comparable at PND 42 and PND 77. These results suggest that mPFC BDNF expression decreased to a similar level from late adolescence to early adulthood in mice.

Given that BDNF is an activity-dependent molecule, previous studies by our group and others have shown that early stress (maternal separation) may disturb the normal developmental pattern of PFC BDNF and its later function (Roceri et al., 2004; Lee et al., 2012; Wang et al., 2015). We found that social stress during early adolescence induced a transient increase in mPFC BDNF mRNA levels 1 week after the last exposure at PND 45, while a long-term decrease in mPFC BDNF mRNA levels was observed 6 weeks later, at PND 87, suggesting a more obvious decline trajectory in previously stressed mice compared to the normal developmental decline tendency from late adolescence to early adulthood in CON mice, as described above. Coppens et al. (2011) reported that social defeat in adolescent rat led to up-regulation of BDNF mRNA expression in hippocampus, but not in the PFC. This discrepancy may be attributable to different experimental conditions in the two studies, including stress duration and age at exposure to stress (Bath et al., 2013). Furthermore, western blot and immunohistochemical analyses also showed a protracted effect of adolescent social stress on BDNF protein expression in the mPFC. This effect was region-specific, given that the decrease in BDNF expression was evident in the mPFC but not in the OFC, as shown by immunohistochemical data. Consistently, human adults who have experienced abuse during childhood and adolescence demonstrate an increased susceptibility to the development of depression and also present with lower salivary BDNF levels (Grassi-Oliveira et al., 2008). It has been shown that BDNF and its receptor TrkB are critically involved in the modulation of neural development and structural and synaptic plasticity in the mPFC (Ward and Hagg, 2000; Finsterwald et al., 2010). Long-term inhibition of BDNF signaling in the rodent PFC by genetic and stress manipulations can alter the pattern of neural connectivity, the growth and complexity of dendrites and synaptic plasticity, and these effects can be reversed by direct BDNF rescue (Lu et al., 2008; Sakata et al., 2013). Thus, the long-term decrease in BDNF expression in adult mice subjected to adolescent social defeat might exert detrimental effects on mPFC plasticity and the cognitive performance mediated by this area (Liston et al., 2006; Ragozzino, 2007).

### Effects of Antidepressant Duloxetine on Cognitive and BDNF Alterations in Adult Mice Submitted to Adolescent Social Stress

We demonstrated that chronic treatment with the antidepressant duloxetine improved the cognitive dysfunction in EDS and the decreased expression of BDNF in the mPFC of previously stressed adult mice. Clinical evidence has demonstrated that the improvement of depressive symptoms and the recovery of neuroplasticity decrease usually occur concomitantly after chronic antidepressant treatment (usually 2~4 weeks; Pompili et al., 2013). Consistent with this, BDNF has emerged as a critical mediator for the efficacy of various antidepressants by reversing the reduced neuroplasticity in the PFC and hippocampus in depression; the same improvement in neuroplasticity also occurs after chronic antidepressant treatment (Castrén and Rantamäki, 2010; Duman and Monteggia, 2006). For example, chronic duloxetine administration increases PFC BDNF mRNA and protein levels in animal models for depression, correlating with an improvement in emotional behaviors (Calabrese et al., 2007; Mannari et al., 2008; Engel et al., 2013). Chronic but not acute duloxetine treatment can also increase BDNF levels in the blood of patients with depression (Fornaro et al., 2015; Hashimoto, 2015). Furthermore, such effects of antidepressant treatments can be prevented by the abolishment or inhibition of BDNF signaling (Adachi et al., 2008). Thus, our study provides extensive evidence that chronic duloxetine treatment during adulthood can rescue the impairment in cognitive flexibility and the decrease in mPFC BDNF expression induced by adolescent social stress.

In summary, our study demonstrates that adolescent social stress impaired cognitive flexibility in adulthood. Moreover, we show that this cognitive dysfunction was accompanied by reductions in the mRNA and protein levels of BDNF in the mPFC. The long-term cognitive and molecular alterations following adolescent stress could be reversed by chronic antidepressant treatment during early adulthood. These findings suggest that adolescent social stress could be used to model depression-related psychopathologies during adulthood, including cognitive impairment and inhibition of BDNF signaling in the mPFC. The identification of molecular targets that can enhance BDNF signaling in the mPFC may reveal the mechanisms underlying cognitive dysfunction and contribute to effective strategies to improve the treatment of depression.

## Author Contributions

WW designed the research; SY, FZ, YZ, and HX performed the research and acquired the data; FZ and WW interpreted and analyzed the data; and HX, YZ, FS and WW drafted, revised and wrote the paper.

## Conflict of Interest Statement

The authors declare that the research was conducted in the absence of any commercial or financial relationships that could be construed as a potential conflict of interest.
